# IFI44L expression is regulated by IRF‐1 and HIV‐1

**DOI:** 10.1002/2211-5463.13030

**Published:** 2020-11-27

**Authors:** Yutong Li, Junshi Zhang, Chenchen Wang, Wentao Qiao, Yue Li, Juan Tan

**Affiliations:** ^1^ Key Laboratory of Molecular Microbiology and Technology Ministry of Education College of Life Sciences Nankai University Tianjin China; ^2^ Department of Microbiology and Immunology Western University London ON Canada

**Keywords:** HIV‐1, IFI44L, interferon, ISRE, promoter

## Abstract

Interferon (IFN)‐inducible 44 like (IFI44L) is an IFN‐stimulated gene (ISG), which is located on the same chromosome as the known antiviral ISG IFI44. Expression of IFI44L is induced by IFN and HIV‐1 infection. However, the mechanism by which IFN‐I induces IFI44L production has not yet been determined. In this study, we analyzed transcriptional regulation of IFI44L via cloning of the *IFI44L* promoter. We found that *IFI44L* has two IFN‐stimulated response elements (ISRE), which are necessary for the basal level of *IFI44L* transcription. IFN‐I and IFN‐II can activate the *IFI44L* promoter through one of the two ISREs. IFN regulatory factor (IRF)‐1 can activate transcription of *IFI44L* by binding to one of the ISREs. Additionally, co‐transfection of the *IFI44L* promoter with an HIV‐1 infectious clone or HIV‐1 infection activated *IFI44L* promoter transcription, but did not upregulate *IFI44L* expression via ISREs. These findings will help to understand the interaction between IFI44L and HIV‐1, and aid in elucidation of the role of IFI44L in the antiviral innate immune response.

## Graphical abstract

Interferon (IFN)‐inducible 44 like (IFI44L) is an IFN‐stimulated gene, the expression of which is induced by IFN and human immunodeficiency virus (HIV)‐1 infection. However, the mechanism has not yet been determined. In this study, we cloned the promoter of the *IFI44L* gene and found that interferon regulatory factor‐1 could bind directly to the IFN‐stimulated response element in the *IFI44L* promoter to activate IFI44L. Furthermore, we demonstrated that HIV‐1 can activate the *IFI44L* promoter to influence the expression of IFI44L.

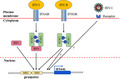

**Abbreviations**
IFI44L: Interferon inducible 44 like
IFN: interferon
IRF: interferon regulatory factor
ISG: interferon‐stimulated gene
ISRE: IFN‐stimulated response element
STAT: signal transducer and activator of transcription

Interferon (IFN)‐inducible 44 like (IFI44L) is a part of the IFI44 family and is located on the same human chromosome as the previously identified antiviral interferon‐stimulated gene (ISG) IFI44, which is involved in numerous signaling pathways of the innate immune response. At the same time, IFI44L is also an ISG and can be induced by many different viruses such as influenza and the HIV‐1 [1, 2]. HIV‐1 proteins can affect the expression of IFI44L through different regulatory pathways in different cell types. For example, HIV‐1 gp120 can downregulate the expression of IFI44L by interaction with α4β7 [3]. Also, the transcription level of IFI44L is significantly upregulated in HIV‐1 gp120‐treated vaginal epithelial cells [4]. Additionally, HIV‐1 Vpr can upregulate the expression of IFI44L [5]. However, the mechanism by which HIV‐1 induces IFI44L up‐regulation has not been determined. In addition to HIV‐1, when rhesus monkeys are infected by the simian immunodeficiency virus, IFI44L is significantly upregulated up to 38‐fold on the 10th day of infection, and the up‐regulation of IFI44L is accompanied by up‐regulation of ISGs [6].

There are three types of IFNs: IFN‐I, IFN‐II, and IFN‐Ⅲ, which participate in a variety of biological activities, including antiviral response, antitumor, inflammatory response, and immunomodulatory activity [7, 8, 9]. IFNs regulate transcription of ISGs through the Janus kinase/signal transducer and activator of transcription (STAT) pathway [10]. Using the IFN‐I signaling pathway as an example, after IFN binds to its receptor, the receptor‐associated tyrosine kinases Tyk2 and Jak1 are activated, and then, STAT1 and STAT2 are phosphorylated to form a heterodimer complex and translocated to the nucleus. The complex is assembled with interferon regulatory factor (IRF)‐9 and binds to the IFN‐stimulated regulatory element (ISRE) on the ISG promoter to induce ISGs transcription [11, 12, 13, 14, 15].

Interferon regulatory factors participate in the IFN‐mediated signaling pathway. Nine members of the IRFs family have been identified in human cells. For example, IRF‐1 can bind the promoter region of IFN‐β and activate transcription. IRF‐2 can also bind to the IFN‐β promoter, but it inhibits IFN‐β expression [16]. IRF3 plays a major role in the transcription of the gene encoding IFN‐1. IRF‐5 plays a role in the immune response against viral infections. IRF‐7 can bind to MyD88 and TRIF and increases the production of IFN‐I induced by the stimulation of some TLRs [9, 17, 18, 19].

The expression of IFI44L in HIV‐1 progressor blood samples are significantly higher than that in nonprogressors, and IFI44L levels are upregulated by IFN‐α [2]. However, the mechanism by which IFN‐I induces IFI44L production is not yet determined. In this study, we cloned the promoter of *IFI44L* gene to better understand its transcriptional regulation. We found IRF‐1 could bind directly to the ISRE in the *IFI44L* promoter to activate IFI44L. Furthermore, we demonstrated that HIV‐1 can activate the *IFI44L* promoter to influence the expression of IFI44L.

## Materials and methods

### Constructs and antibodies

Plasmids pGL‐1190, pGL‐695, pGL‐390, pGL‐274, pGL‐143, pGL‐117‐1190, pGL‐237‐1190, pGL237‐390, and pGL380‐1190 were constructed by cloning the PCR‐amplified fragment of the IFI44L promoter into pGL3‐basic (Promega, Madison, WI, USA), primers are listed in Table 1. pGL‐274mISRE‐1 and pGL‐237‐1190mISRE‐2 were constructed using site‐directed mutagenesis kit (Toyobo, Osaka, Japan), and primers used are listed in Table 1. Plasmids IRF‐1, IRF‐2, IRF‐3, IRF‐5, and IRF‐7 were constructed by inserting the CDS into the pCDNA3.1 (+) (Invitrogen, Carlsbad, CA, USA) or pCMV‐Tag3B (Stratagene, La Jolla, CA, USA). Sequences of all constructs were confirmed by sequencing.

**Table 1:** Primers for amplifying *IFI44L* promoter fragments and site‐directed mutagenesis.

Primer	Sequence (5′–3′)
p1190	GAGCCGCCTTAATAACTTCTC
p695	GCCTCGGCAGAGACGACCCAGGAGA
p390	CGACGCGTGCCTGCCTACATACATAC
p274	CGACGCGTCTGCCAGCTGAGTTTTTTTGC
p143	CGACGCGTCTTTCTTTCCTAGAGTCTCTG
p117‐1190‐R	CCCTCGAGGGCTTCAGAGACTCTAGGAAA
p237‐1190‐R	CCCTCGAGCCTGAAACTCAAAGCAGCAA
p380‐1190‐R	CCCTCGAGGGCAGGCATGAAATGATAAC
mISRE‐F	CTCTTCCTAGTGAGGACAAAGACAGTTAGTGGCAGTTG
mISRE‐R	CAACTGCCACTAACTGTCTTTGTCCTCACTAGGAAGAG

Antibodies against Myc, IRF‐1, IRF‐2, Tubulin, IgG, and RNA Polymerase were purchased from Sigma (St. Louis, MO, USA), and antibody against IFI44L was purchased from Aviva (San Diego, CA, USA).

### Cell culture and transfection

293T, HeLa, and TZM‐bl cells (maintained in our lab) were grown in Dulbecco's Modified Eagle's medium (Gibco, Gaithersburg, MD, USA) medium supplemented with 10% FBS (BI) in 5% CO_2_ at 37 °C. Jurkat cells were grown in RPMI 1640 (Gibco) medium supplemented with 10% FBS (BI) in 5% CO_2_ at 37 °C. Transfection was performed using Polyetherimide reagent (Sigma‐Aldrich) and Lipo3000.

### HIV‐1 pseudovirus preparation

The HIV‐1 pseudovirus was prepared by transfecting 293T cells with NL4‐3 and pVSV‐G. To prepare cell‐free HIV‐1 stocks, culture supernatants were cleared by low‐speed centrifugation (3000 ***g*** for 10 min), filtered through a 0.22‐μm‐pore‐size filter membrane, and kept at 4 °C. HIV‐1 titers were determined by infecting TZM‐bl cells.

### Chromatin immunoprecipitation (ChIP)

ChIP analysis was conducted based on manufacturer's instructions using EZ‐chip kit (Millipore, Billerica, MA, USA). PCR primers used for amplifying the IFI44L promoter are as follows: forward, 5′ CAAGGGGACCAGTGATAG‐3′ and reverse: 5′‐GATCTGTGGCTTCAGAGACTC‐3′.

### Western blot

Cells were washed with PBS and lysed in lysis buffer [20 mm Tris (pH 7.4), 150 mm NaCl, 3% glycerol, 0.25% sodium deoxycholate, 1% NP‐40, complete protease inhibitor cocktail tablets (Roche, Basel, Switzerland)]. Cell lysates were applied to 10% SDS/PAGE and subsequently blotted onto Polyvinylidene fluoride membrane (GE Healthcare, Little Chalfont, Buckinghamshire, UK). The membranes were blocked and incubated with primary antibodies and then with HRP‐conjugated secondary antibodies. Enhanced chemiluminescence detection reagents (Millipore) were used for signal detection.

### Luciferase reporter assay

HeLa cells were transfected with luciferase plasmids and β‐galactosidase expression plasmids. Luciferase activity was determined with luciferase report system (Promega) and normalized to β‐galactosidase activity.

## Results

### Promoter activity analysis of *IFI44L*

**Fig. 1:**
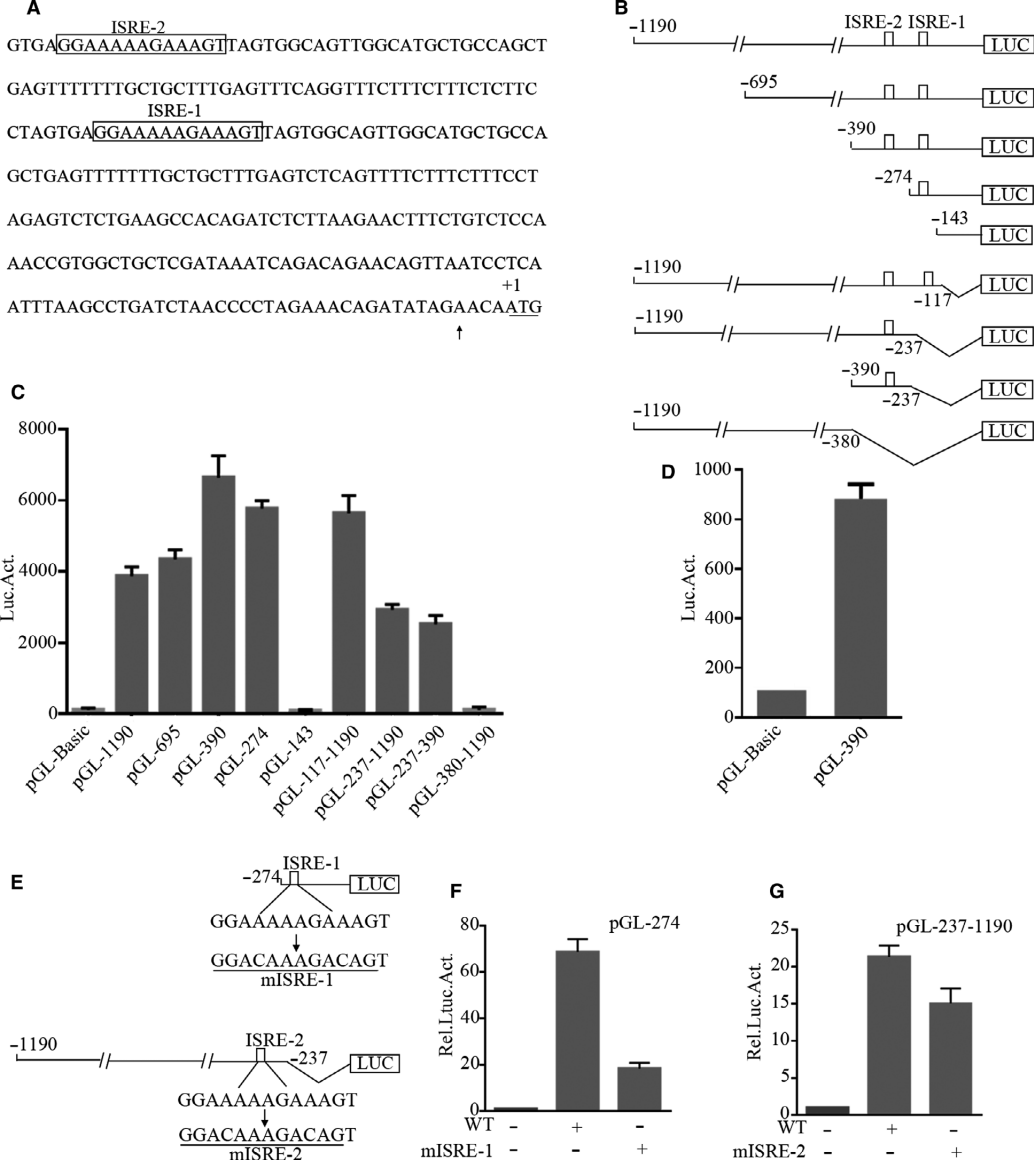
Identification and analysis of *IFI44L* promoter. (A) Partial sequence of *IFI44L* promoter. Arrow indicates the starting point of transcription and the A is designed as +1. The potential cis‐elements are boxed. (B) The 5′‐truncated plasmids of *IFI44L* promoter. (C) The promoter constructs (200 ng) were transfected into HeLa cells (1 × 10^5^). Forty‐eight hours post‐transfection, luciferase assay was performed, and β‐galactosidase activity was used as a normalization control for the luciferase activity. (D) The pGL‐390 or pGL‐Basic (600 ng) was transfected into Jurkat cells (1 × 10^6^). Forty‐eight hours post‐transfection, luciferase assay was performed, and β‐galactosidase activity was used as a normalization control for the luciferase activity. (E) Mutations of ISRE on pGL‐274 and pGL‐237‐1190, respectively. (F) The wild‐type or mutant pGL‐274 and (G) pGL‐237‐1190 or mutant pGL‐237‐1190 (200 ng) were transfected into HeLa cells (1 × 10^5^), and luciferase was detected 48 h post‐transfection. The results shown represent the averages of the results of three independent experiments. Error bars indicate SD.

To clarify the mechanism how *IFI44L* gene is induced by IFNs, we first studied the transcriptional regulation mechanism of IFI44L. We used the translation start site (ATG) as +1 and determined that the first exon of *IFI44L* is located at −16 to −183 bp through comparing the *IFI44L* genomic sequence in UCSU, Ensembl, and the *IFI44L* mRNA sequence in NCBI. Then, we cloned the 1190 bp DNA upstream of the transcription initiation site and performed sequence analysis. The results showed that it contained two ISRE components (Fig. 1A). To verify whether the *cis*‐acting elements are involved in the transcription, a series of deletions of the promoter were constructed (Fig. 1B). We transfected these reporter plasmids into HeLa cells and measured the luciferase activity 48 h post‐transfection. As shown in Fig. 1C, the basal transcriptional activity of truncated *IFI44L* promoter from +1 to −274 (with the ISRE‐2 deleted) decreased moderately, while the basal transcriptional activity of the *IFI44L* promoter was further reduced when the truncation spanned position −237 to −1190 (with the ISRE‐1 deleted). In addition, we also studied the basal transcriptional activity of pGL‐390 in Jurkat cells to ensure the reliability of the above results, and similar results were obtained (Fig. 1D). In order to further confirm whether the two ISREs are involved in regulating the basic transcriptional activity of the *IFI44L* promoter, we constructed a series of mutations on pGL‐274 and pGL‐237‐1190 (Fig. 1E). As shown in Fig. 1F,G, the basal transcriptional activity decreased by 72% (ISRE‐1 mutation) and 14% (ISRE‐2 mutation). Furthermore, when the two elements were mutated, pGL‐143 completely lost the promoter activity compared to the empty vector. Therefore, we concluded that the two ISREs are essential elements in the basal transcription of *IFI44L*.

### The ISRE‐1 element is essential for IFNs to activate *IFI44L* promoter

**Fig. 2:**
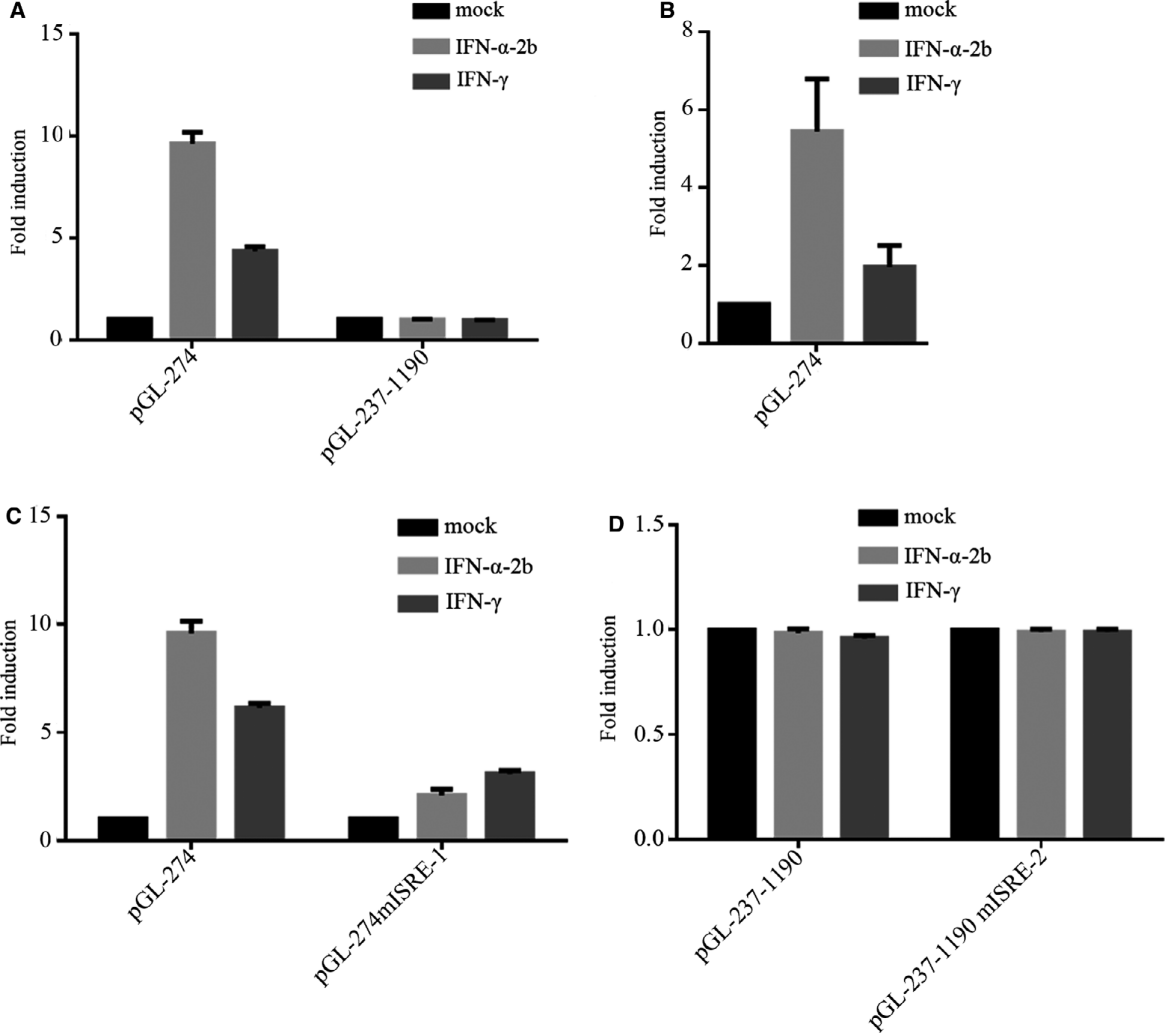
IFNs activate *IFI44L* transcription via ISRE‐1. (A) pGL‐274 or pGL‐237‐1190 (200 ng) was transfected into HeLa cells (1 × 10^5^). Thirty‐six hours post‐transfection, the cells were treated with IFN‐α or IFN‐γ (10 ng·mL^−1^) for 12 h. Determination of luciferase activity after 48 h transfection. (B) pGL‐274 (600 ng) was transfected into Jurkat cells (1 × 10^6^). Thirty‐six hours post‐transfection, the cells were treated with IFN‐α or IFN‐γ (10 ng·mL^−1^) for 12 h. Determination of luciferase activity after 48‐h transfection. (C and D) pGL‐274, pGL‐274mISRE‐1 or pGL‐237‐1190, pGL‐237‐1190mISRE‐2 (200 ng) were transfected into HeLa cells (1 × 10^5^), and then, cells were stimulated with IFN‐α or IFN‐γ (10 ng·mL^−1^) 12 h before luciferase detection. The results shown represent the averages of the results of three independent experiments. Error bars indicate SD.

To verify whether the *IFI44L* promoter responds to IFNs, HeLa cells were transfected with pGL‐274 or pGL‐237‐1190; 36h post‐transfection, cells were treated with IFN‐α or IFN‐γ for 12 h, respectively. As shown in Fig. 2A, both IFN‐α and IFN‐γ could activate pGL‐274 (with ISRE‐1). Besides, we also transfected pGL‐274 into Jurkat cells and treated with IFNs, and got similar results as 2A (Fig. 2B). Furthermore, IFN‐α was more potent than IFN‐γ at inducing promoter response. However, IFN‐α and IFN‐γ could not activate pGL‐237‐1190 (with ISRE‐2). To further confirm whether IFNs activate pGL‐274 via the ISRE‐1, we constructed ISRE‐1 mutant plasmids (pGL274mISRE‐1). We found that the *IFI44L* promoter did not respond to IFNs when the ISRE‐1 was mutated (Fig. 2C,D). These results demonstrated that the ISRE‐1 is essential for IFNs to activate the *IFI44L* promoter.

### IRF‐1 binds to the ISRE to increase *IFI44L* expression

**Fig. 3:**
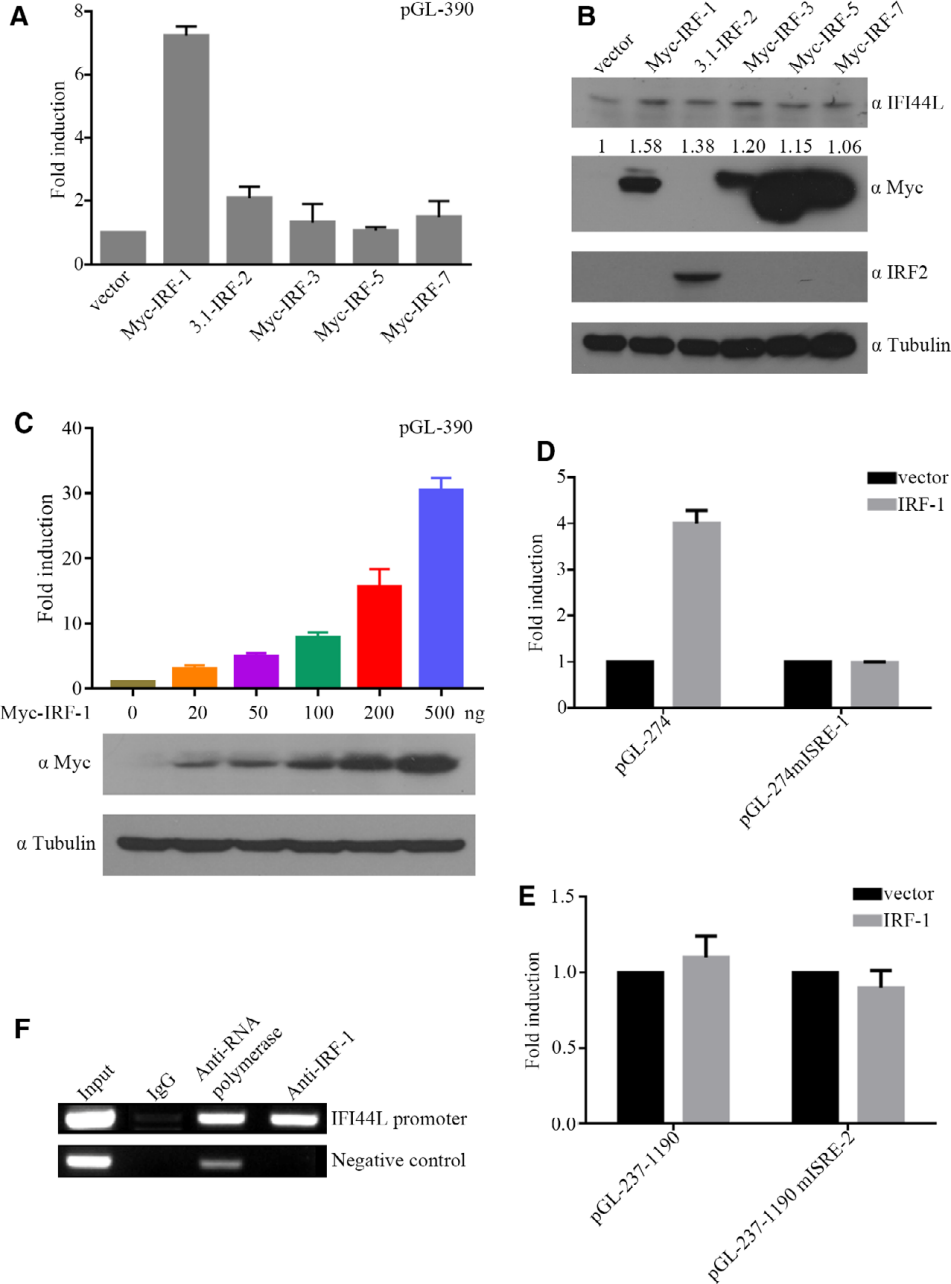
IRF‐1 binds to the ISRE‐1 to activate *IFI44L* promoter. (A and B) pGL‐390 (200 ng) with IRFs (400 ng) transfected into HeLa cells (1 × 10^5^), 48 h post‐transfection, luciferase activity and western blot analysis was performed. (C) 293T cells (2 × 10^5^) were transfected with 200 ng pGL‐390 and 20, 50, 100, 200, 500 ng IRF‐1. (D) pGL‐274, pGL‐274mISRE‐1 (200 ng) or (E) pGL‐237‐1190, pGL‐237‐1190 mISRE‐2 (200 ng) were co‐transfected with pcDNA3.1 or pcDNA3.1‐IRF‐1 (400 ng) into HeLa cells (1 × 10^5^). Luciferase activity was measured 48 h after transfection. (F) HeLa cells (1 × 10^5^) were treated with IFN‐α (10 ng·mL^−1^) for 12 h and detected with anti IRF‐1, control IgG, and anti‐RNA polymerase. Precipitated DNA was amplified by PCR with primers designed for *IFI44L* ISRE (up) or GAPDH promoter (down). The results shown represent the averages of the results of three independent experiments. Error bars indicate SD.

The IRF family can participate in the transcriptional regulation of the target genes. In order to identify whether the IRF family is involved in regulating *IFI44L*, we co‐transfected Myc ‐IRF‐1, 3.1‐IRF‐2, Myc‐IRF‐3, Myc‐IRF‐5, Myc‐IRF‐7 encoding plasmids with pGL‐390 into HeLa cells and measured the transcription activity of pGL‐390. As shown in Fig. 3A,B, overexpression of IRF‐1 significantly activated pGL‐390 in HeLa cells, the trend measured by western blot was consistent with luciferase results, but protein levels were not as sensitive as transcriptional levels. In order to further confirm that IRF‐1 can upregulate the *IFI44L* promoter, we co‐transfected the *IFI44L* promoter and dose‐gradient Myc‐IRF‐1 into 293T cells, and found that *IFI44L* promoter could be upregulated by IRF‐1 in a dose‐dependent manner (Fig. 3C). We then further explored whether IRF‐1 could activate the *IFI44L* promoter via the ISRE element. We co‐transfected Myc‐IRF‐1 and pGL‐274mISRE‐1 or pGL‐237‐1190mISRE‐2 into HeLa cells. As shown in Fig. 3D,E, IRF‐1 did not activate the pGL‐274mISRE‐1, indicating that IRF‐1 activated the *IFI44L* promoter via the ISRE‐1 element. Then we examined whether endogenous IRF‐1 could also interact with ISRE when stimulated by IFN. ChIP assays were performed after treating HeLa cells with IFN‐α and followed by PCR. The results showed that endogenous IRF‐1 could interact with the ISRE of *IFI44L* (Fig. 3F). The results above indicate that IRF‐1 can bind to the ISRE of *IFI44L* promoter to regulate *IFI44L* expression.

### HIV‐1 can activate *IFI44L* promoter

**Fig. 4:**
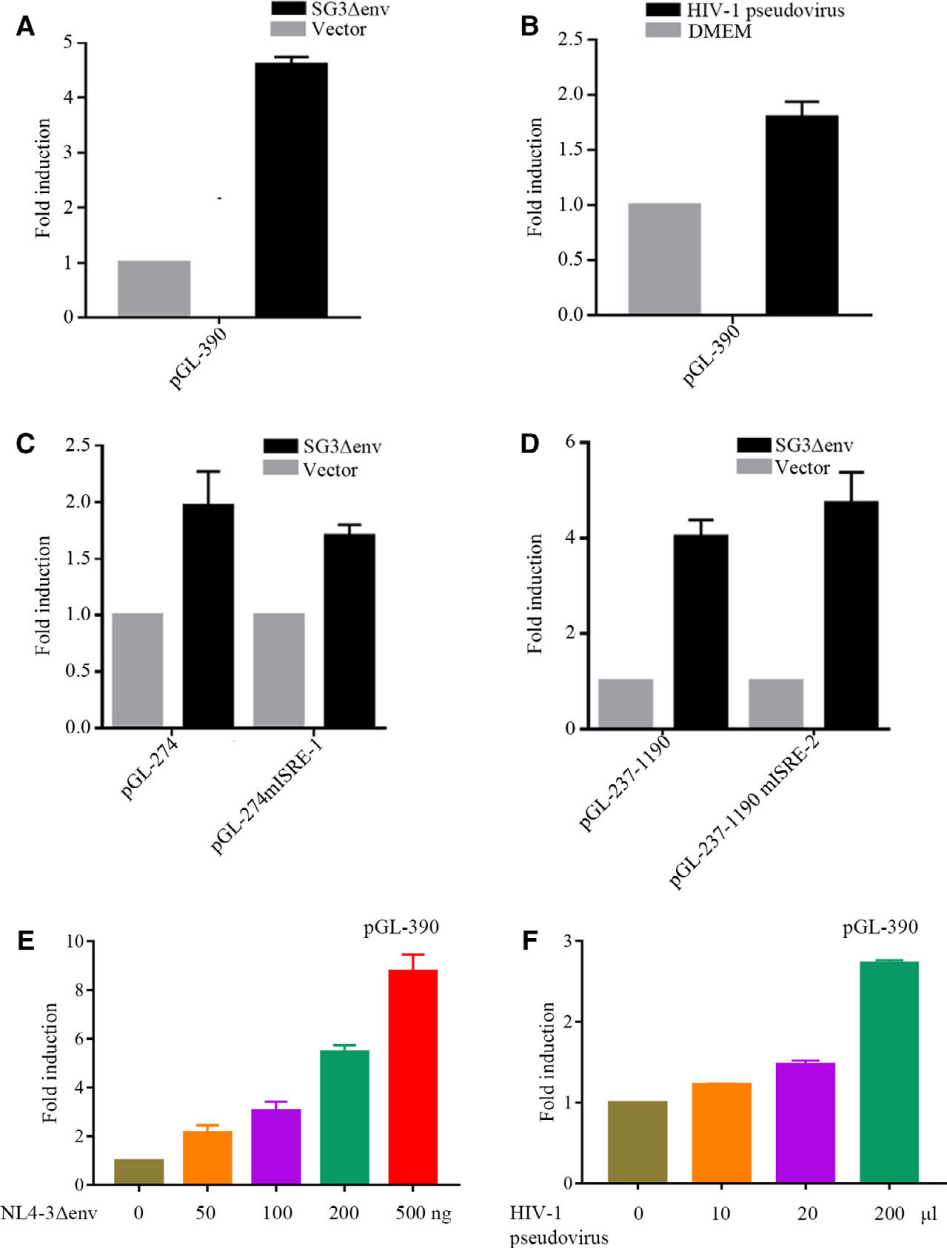
HIV‐1 activates *IFI44L* promoter. (A) 293T cells (2 × 10^5^) were transfected with SG3Δenv (500 ng) and pGL‐390 (200 ng), 48 h post‐transfection, luciferase assays were performed. (B) 293T cells (2 × 10^5^) were transfected with pGL‐390 (200 ng); after 8 h, cells were infected with HIV‐1 pseudovirus. Luciferase assays were performed. (C, D) 293T cells (2 × 10^5^) were transfected with SG3Δenv (500 ng) and pGL‐274, pGL‐274 mISRE‐1 (200 ng) or pGL237‐1190, pGL‐237‐1190mISRE‐2 (200 ng), 48 h post‐transfection, luciferase assays were performed. (E) 293T cells (2 × 10^5^) were transfected with 200 ng pGL‐390 and 50, 100, 200, 500 ng NL4‐3Δenv; 48 h post‐transfection, luciferase assays were performed. (F) 293T cells (2 × 10^5^) were transfected with 200 ng pGL‐390, after 8 h, cells were infected with 10, 20, 200 μL HIV‐1 pseudovirus. Luciferase assays were performed. The results shown represent the averages of the results of three independent experiments. Error bars indicate SD.

To determine whether HIV‐1 can upregulate *IFI44L* promoter activity, we co‐transfected SG3Δenv and pGL‐390 into 293T cells. As shown in Fig. 4A,B, HIV‐1 can upregulate *IFI44L* promoter activity. Furthermore, co‐transfecting SG3Δenv and pGL‐274, pGL237‐1190, pGL‐274mISRE‐1, pGL‐237‐1190mISRE‐2 into 293T cells, HIV‐1 did not directly interact through ISRE elements to upregulate *IFI44L* promoter activity (Fig. 4C,D). To further confirm that HIV‐1 can upregulate *IFI44L* promoter, we co‐transfected 293T cells with dose‐gradient NL4‐3Δenv and pGL‐390, or infected 293T cells with HIV‐1 pseudovirus with dose gradient after pGL‐390 transfection. The results showed that HIV‐1 can upregulate *IFI44L* promoter in a dose‐dependent manner (Fig. 4E,F).

## Discussion

Interferons can activate the expression of ISGs through the JAK‐STAT pathway, which inhibit viruses at different stages of replication. HIV‐1 is able to regulate a number of ISGs to ensure its own infection [20, 21]. As an ISG, IFI44L can be induced by IFNs and HIV‐1. In this study, we explored the mechanism of how IFI44L is induced by IFNs and HIV‐1.

First, we cloned and analyzed the features of the *IFI44L* promoter and we found that the *IFI44L* promoter has two ISREs, but no TATA and GC boxes. Two ISRE components are located in the complete repeat sequence [22, 23]. We identified that the basal transcriptional activity of *IFI44L* is significantly decreased when the ISREs are mutated, indicating that these two ISRE elements are essential for the transcriptional regulation of *IFI44L*, especially ISRE‐1. This is similar to some other ISGs, which are controlled by ISRE. After IFN‐I stimulates cells, a heterodimer complex is formed by phosphorylation of STAT1 and STAT2, which is assembled with IRF‐9 and induces ISGs production by binding to ISRE [11, 12, 13, 14, 15]. Furthermore, we determined that IFN‐I can activate *IFI44L* promoter and that it is through ISRE‐1 rather than ISRE‐2. IFN‐Ⅱ can also induce up‐regulation of *IFI44L* promoter activity, but the upregulate level is lower than that of IFN‐I. We suspect that this upregulation of IFN‐II is indirect because we cannot be sure that there is a gamma‐interferon activation site element on the *IFI44L* promoter.

IFN regulatory factors are a class of transcriptional regulatory proteins that regulate the expression of IFNs and ISGs. Therefore, we also tested whether IRFs could activate *IFI44L*. We observed that IRF‐1 can significantly activate the transcription of *IFI44L*. This is because IRF‐1 can recognize ISRE and activate the transcription of IFN‐I and ISGs [24, 25], which indicates that IFI44L may participate in the host's innate immunity through these IRFs.

As mentioned earlier, HIV‐1 proteins can affect the expression of IFI44L through different regulatory pathways in different cell types. Furthermore, we demonstrated that HIV‐1 can affect the expression of IFI44L by upregulating the activity of the *IFI44L* promoter in 293T cells. Besides, Lu *et al*. [26] reports that high‐throughput screening experiments at the cellular level have detected a significant increase in HIV‐1 replication levels after IFI44L has been knocked out. We speculated that IFI44L plays an immunoregulatory role for HIV‐1 in host cells, one possible reason is that HIV‐1 can ensure latent infection by up‐regulating the expression of IFI44L. However, after mutation of ISRE, HIV‐1 can still upregulate the *IFI44L* promoter activity, therefore, HIV‐1 did not pass the ISRE to affect the *IFI44L* promoter activity. The mechanism by which HIV upregulates the promoter activity of *IFI44L* needs further study.

In summary, this study found that IFN‐I and IFN‐II can upregulate the transcription of the *IFI44L* promoter, and HIV‐1 can activate the *IFI44L* promoter to influence the expression of IFI44L. We will continue to explore the specific mechanism by which HIV‐1 upregulates IFI44L initiation activity. This will help to understand the more physiological functions of IFI44L and the interaction between IFI44L and HIV‐1, and determine the status of IFI44L in antiviral innate immune response.

## Conflict of interest

The authors declare no conflict of interest.

## Author contributions

YL, JT, YL, and JZ designed the study. YL and JZ drafted the manuscript, and JT and YL helped modify the manuscript. YL, JZ, and CW performed the experiments. YL and JZ contributed equally to the work. All authors read and approved the final manuscript.

## Data Availability

Data will be available from the corresponding author upon reasonable request.
